# Microsporidial Keratoconjunctivitis after Rugby Tournament, Singapore

**DOI:** 10.3201/eid1909.121464

**Published:** 2013-09

**Authors:** Junda Tan, Phoebe Lee, Yingqi Lai, Pengiran Hishamuddin, Joanne Tay, Ai Ling Tan, Kian Sing Chan, Raymond Lin, Donald Tan, Jeffery Cutter, Kee Tai Goh

**Affiliations:** Ministry of Health, Singapore (J. Tan, P. Lee, Y. Lai, P. Hishamuddin, J. Tay, R. Lin, J. Cutter, K.T. Goh);; Singapore General Hospital, Singapore (A.L. Tan, K.S. Chan); Singapore National Eye Centre, Singapore (D. Tan);; Saw Swee Hock School of Public Health, National University of Singapore, Singapore (K.T. Goh)

**Keywords:** Microsporidia, Vittaforma corneae, keratoconjunctivitis, parasites, fungi, Singapore

## Abstract

We investigated an outbreak of 47 probable and 6 confirmed cases of microsporidial keratoconjunctivitis involving participants of an international rugby tournament in Singapore in April 2012.The mode of transmission was eye contact with soil. *Vittaforma corneae* was identified in 4 of 6 corneal scrapings and in 1 of 12 soil water samples.

Microsporidia are spore-forming single-cell intracellular parasites, which have recently been shown to be fungi on the basis of phylogenetic analyses (*1*). They are ubiquitous in the environment, and at least 14 species have been implicated in human infections (*2*). Human ocular microsporidiosis first came into prominence as an opportunistic infection in patients with AIDS in the 1980s and, subsequently, in other immunocompromised patients (*3*,*4*). In the 1990s, *Vittaforma corneae* (formerly known as *Nosema corneum*) (*5*) was first described as the cause of corneal infection in an immunocompetent person (*6*) and disseminated infection in an immunocompromised patient (*7*). Since the early 2000s, microsporidial keratoconjunctivitis has been increasingly reported, mostly in Singapore (*8*–*10*) and India (*11*), among healthy, immunocompetent persons. The infections result predominantly from eye contact with soil or mud in outdoor activities. 

On May 18, 2012, the Ministry of Health, Singapore, received a notification from the Centre for Health Protection, Hong Kong, of a suspected outbreak of microsporidial keratoconjunctivitis that was affecting 18 boys in a rugby club who had participated in an international rugby tournament in Singapore on April 21–22, 2012. We report the epidemiology, clinical features, and laboratory findings of the outbreak.

## The Study

After the notification, epidemiologic investigations were undertaken immediately. A medical alert of the outbreak was circulated to all registered medical practitioners in Singapore. Local case-patients identified by clubs and medical practitioners (including ophthalmologists) were interviewed by telephone or email by using a set of questionnaires to obtain relevant clinical and epidemiologic data such as age, sex, nationality, clinical signs and symptoms, date of onset of illness, medical treatment sought, and details of activities at the tournament (Figure). Corneal scrapings collected by ophthalmologists were tested by microscopy; modified trichrome staining was used to detect microsporidial sporelike structures. Samples demonstrating these structures were then subjected to DNA extraction and microsporidia-specific PCR sequencing following previously described protocols (*12*). Soil water (water mixed in soil found in muddy fields) samples collected from the tournament venue on May 22, 2012, were tested for microsporidia by the Department of Pathology, Singapore General Hospital. The strategy of serial centrifugation (*13*) was adopted, followed by modified trichrome staining to detect microsporidial sporelike structures. Samples showing these structures also underwent microsporidia-specific PCR sequencing for species identification (*14*).

**Figure:**
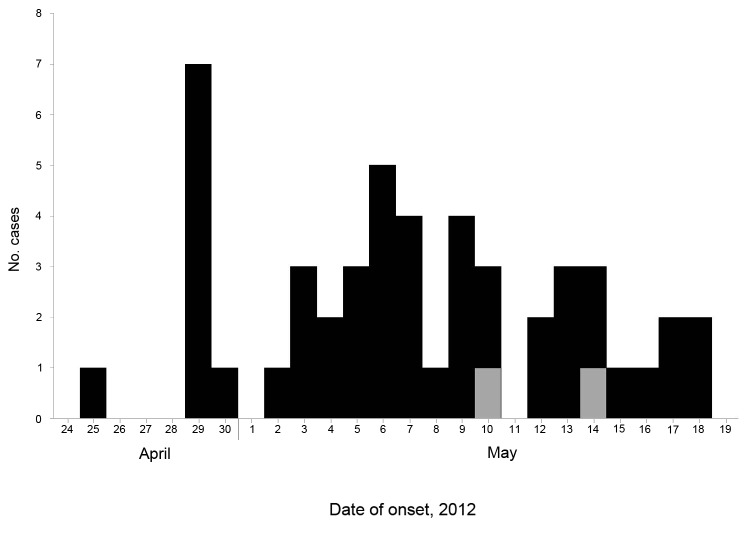
Dates of onset of eye signs and symptoms of microsporidial keratoconjunctivitis in 49 affected local participants after an international rugby tournament in Singapore, April 21–22, 2012. Black indicates probable cases; gray indicates confirmed cases. ND, not determined.

To investigate possible infections in participants from outside Singapore, we contacted the International Health Regulations National Focal Points of the countries involved and team representatives of foreign rugby clubs. We obtained information on participants in whom symptoms of eye infection developed after the tournament.

The rugby tournament involved 1,594 players of 107 teams from rugby clubs from Singapore, Hong Kong, Malaysia, Australia, and the United Arab Emirates. Tournament participants comprised 1,511 boys and 83 girls; Singapore clubs were represented by 1,122 male players and 69 female players. Two types of rugby were played: touch rugby and full-contact rugby.

A probable case of microsporidial keratoconjunctivitis in a person was defined as follows: ≥2 of the following eye signs or symptoms—redness, pain/foreign body sensation, itch, blurred vision, photosensitivity, and/or epiphora—developing from 2 to 30 days after the person participated in the rugby tournament plus a clinical diagnosis by an ophthalmologist using slit lamp biomicroscopy. Biomicroscopy typically revealed the classic microsporidial corneal infection— coarse, multifocal, granular punctuate epithelial keratitis, along with mild follicular or papillary conjunctivitis. The case was classified as confirmed if corneal scrapings were collected and microsporidial spores were shown by microscopy and modified trichrome staining.

Of the 72 local players traced and interviewed, we identified 48 case-patients (46 probable and 2 confirmed) among the boys and 1 probable case-patient among the girls. Among foreign participants, 4 confirmed cases were identified (Table 1). Besides these affected players, 5 probable cases among Singapore residents were identified, comprising 2 coaches, 2 spectators, and 1 referee. In addition, 6 probable sporadic cases were also notified during the outbreak period; they were identified in persons who were not linked to the tournament but who had participated in other outdoor activities with exposure to mud. 

**Table 1:** Microsporidial keratoconjunctivitis cases and attack rates among players from 5 participating countries in rugby tournament, Singapore, April 21–22, 2012

Country	Total no. players	No. (%) probable cases	No. (%) confirmed cases	Overall attack rate, no. (%)
Singapore	1,191	47 (3.9)	2 (0.2)	49 (4.1)
Hong Kong	82	0	3 (3.7)	3 (3.7)
Malaysia	281	0	1 (0.4)	1 (0.4)
Australia	19	0	0	0
United Arab Emirates	21	0	0	0
Total	1,594	47 (2.9)	6 (0.4)	53 (3.3)

Forty-six (93.9%) of the 49 affected Singapore players interviewed were children of expatriates. Symptoms developed from April 25 to May 18, 2012. Their ages ranged from 6 years to 17 years (median age, 12 years). The attack rate among full-contact rugby players (5.7%) was significantly higher than that among touch rugby players (0.5%) (p<0.0001) (Table 2).

**Table 2:** Microsporidial keratoconjunctivitis cases and attack rates by type of rugby contact among local players in international rugby tournament, Singapore, April 21–22, 2012

Category	No. players	No. teams	No. (%) probable cases	No. (%) confirmed cases	Overall attack rate, no. (%)
Touch rugby*	369	33	2 (0.5)	0	2 (0.5)
Full-contact rugby†	822	48	45 (5.5)	2 (0.2)	47 (5.7)
Total	1191	81	47 (3.9)	2 (0.2)	49 (4.1)

*For boys 6– 8 years of age and all girls’ teams. †For boys 9–18 years of age.

The main presenting ocular signs and symptoms were redness (49/49 patients, 100%), pain/foreign body sensation (41/49 patients, 83.7%), photosensitivity (38/49 patients, 77.6%), blurred vision (37/49 patients, 75.5%), itching (34/49 patients, 69.4%), and epiphora (23/49 patients, 46.9%). Twelve case-patients had bilateral infection. The median incubation period, based on the interval between date of last exposure and onset of illness, was 15 days (range 3–26 days). None of the case-patients was hospitalized. All case-patients responded well to treatment.

Forty-six (93.9%) of the affected players reported having had mud enter their eyes while playing in the tournament. Of these, 43 (87.8%) did not share any personal articles such as towels and handkerchiefs with other players, 12 (24.5%) used the available shower facilities, and 41 (83.7%) indicated that they had washed their faces with water from water hoses or mineral water bottles after each match.

Laboratory analysis of 3 corneal scrapings collected from 2 affected local players and 1 Malaysian player who sought treatment at a private eye center in Singapore revealed microsporidial sporelike structures by microscopy with modified trichrome staining. One corneal scraping was confirmed as *V. corneae* by PCR sequencing with 97% sequence homology to at least 2 published *V. corneae* sequences. The same species was also identified in 3 affected players from Hong Kong rugby clubs (*15*). Sporelike structures consistent with microsporidia were also detected in 12 of 21 soil water samples (average dimensions 2.87 μm × 1.68 μm). *V. corneae* was detected in 1 soil water sample.

## Conclusions

We describe a single-source microsporidial keratoconjunctivitis outbreak, to which several factors contributed. First, extreme weather (2 days of heavy rain preceding the tournament and on its first day) resulted in the muddy condition of the field. Second, in full-contact rugby, the risk for exposure of the face and eyes to mud and groundwater is high because the defensive players would have to stop the player with the ball by tackling him or her to the ground. Third, the limited washing facilities at the tournament venue resulted in many players having to wash up at home many hours after exposure to mud.

The main limitation of the study is that the majority of the reported cases were not confirmed by laboratory identification of microsporidia. We could not justify obtaining corneal scrapings from the affected players because the participating ophthalmologists became extremely aware of the characteristic signs and symptoms of microsporidial keratoconjunctivitis. In addition, because of the limited amount of clinical materials available for testing for *V. corneae,* no further genetic studies were undertaken to establish their relatedness.

Microsporidial keratoconjunctivitis is an emerging eye infection in Singapore. Public health professionals should be aware that it may be prevalent in other countries when keratoconjunctivitis is considered as a diagnostic possibility. 
